# Cognitive outcomes of transcranial magnetic stimulation in treatment-resistant depression: a randomized controlled study

**DOI:** 10.55730/1300-0144.5580

**Published:** 2022-11-03

**Authors:** Tahir YILDIZ, Nalan Kalkan OĞUZHANOĞLU, Osman Zülkif TOPAK

**Affiliations:** Department of Psychiatry, Faculty of Medicine, Pamukkale University, Denizli, Turkey

**Keywords:** Depression, transcranial magnetic stimulation, neurocognitive functions

## Abstract

**Background/aim:**

Major depressive disorder (MDD) is a significant cause of workforce loss, and is associated with cognitive impairments which can continue even after the elimination of mood and behavioural symptoms. The aim of this study was to investigate the benefit of transcranial magnetic stimulation (TMS) on cognitive functions in treatment resistant depression.

**Materials and methods:**

This randomised controlled clinical trial was conducted at a university hospital, department of psychiatry (tertiary centre) between October 2019 and July 2020. The study included 30 patients with depressive disorder, aged 18–50 years, who did not respond to at least two antidepressant medications for at least 8 weeks (one drug used was serotonin norepinephrine reuptake inhibitor [SNRI]; and 15 healthy control subjects. The patients were separated into two equal groups in a double-blind, random manner, and 20 sessions of repeated TMS was applied to one group, and 20 sessions of sham TMS to the other. The Montgomery Asberg Depression Scale (MADRS), Hamilton Depression Rating Scale (HAM-D), Stroop test, Wisconsin Card Sorting Test (WCST), Digit Span Test (DST), Trail Making Test A-B, and Verbal Memory Processes Test (VMPT) were applied to the patients before and after the TMS procedure.

**Results:**

The decrease in the HAM-D score was greater in the active magnetic stimulation (25 trains, 10 Hz, 110% motor threshold intensity) group, and with the exception of verbal memory processes, better performance was obtained by the active magnetic stimulation group than the sham group in the cognitive function tests.

**Conclusion:**

TMS was seen toimprove the cognitive defects present in the active phase of treatment-resistant depression, and therefore TMS could provide early improvement in cognitive functions in clinical use.

## 1. Introduction

Major depressive disorder (MDD) is a significant cause of workforce loss, and is associated with cognitive disorders and mood and behavioural symptoms [1,2]. Cognitive losses seen in patients are characterised by one or more defects in cognitive areas such as working memory and attention, with a negative cognitive bias to social conditions or other environmental stimuli [3]. Published data have shown that the cognitive defects observed during MDD continue even after the elimination of mood and behavioural symptoms [4–6].

The cognitive defects seen in MDD are associated with difficulties in activities of daily living, a negative effect on functionality, a decrease in work performance, and reduced productivity in the workplace [3,6,7]. Previous studies have demonstrated that cognitive dysfunction seen in patients with MDD, including the first episode, continues to be present despite optimal symptom control and remission [8–10].

Although there is no common definition of treatment-resistant depression, the most accepted definition is the condition in which “there is no response to at least two different and appropriate antidepressant treatments applied at a sufficient dose and duration” [11]. Repeated transcranial magnetic stimulation (rTMS) is one of the alternative procedures that can be used to treat resistant depression [12–14]. The ability of TMS to regulate cortical stimulation suggests that it could be a useful tool for the reshaping of cortical networks which will also change cognitive performance [15]. As cognitive deficits in depression constitute a notable component of the disease burden, drug studies on this subject have accelerated in recent years (such as vortioxetine). However, drug treatments have some side-effects, and therefore, safer treatments are needed. TMS is a noninvasive brain stimulation technique for neuromodulation and is especially targeted on the glutamatergic system [16]. Recent studies have shown that TMS improves cognitive functions in areas such as concentration, executive functions, working memory and long-term verbal memory [17]. There are also studies showing that it may be an effective treatment in mild cognitive disorder, even in the elderly, and could delay a worsening of the condition [18].

The aim of the current study was to investigate whether or not TMS had a regulatory effect on cognitive disorders in treatment-resistant depression.

## 2. Materials and methods

### 2.1. Participants

The study designed as a randomised controlled trial; included 30 patients, aged 18–50 years, who were diagnosed with MDD according to the Diagnostic and Statistical Manual of Mental Disorders, Fifth Edition (DSM-5) criteria as outpatients in the Psychiatry Department of Pamukkale University Faculty of Medicine between October 2019 and July 2020. All the patients had full mental capacity, were literate, had no physical or neurological disease, had not responded to at least two antidepressant medications, one of which was serotonin norepinephrine reuptake inhibitor (SNRI) administered for at least 8 weeks, had no psychotic findings, and were not at severe risk of suicide. A control group was formed of 15 healthy volunteers, matched to the patient group in respect of age, sex and smoking status, and with no familial history of psychiatric disease.

The patients were separated into two equal groups in a double-blind, random manner. In one group, 20 sessions of high frequency (10Hz) rTMS were applied to the left dorsolateral prefrontal cortex, and in the other group, 20 sessions of sham TMS. Throughout the study, all the patients continued taking their pharmacological treatments at the same dose. The calculated power (1-beta) was 0.72; considering type 1 error (alfa) of 0.05, sample size of 15, and effect size of 0.44’.

### 2.2. Measurement tools

For all the study participants, a sociodemographic data form was completed, and the structured clinical interview form for DSM (SCID-5) was applied [19]. Before and after the procedures, the patient groups were applied with Montgomery-Asberg Depression Scale (MADRS) [20], Hamilton Depression Inventory (HAM-D) [21], Wisconsin Card Sorting Test (WCST) [22], Stroop test [23], Trail Making Test A-B (TMT A-B) [24], Digit Span Test (DST) [22], and Verbal Memory Process Test (VMPT) [25].

Informed consent was obtained from all the participants, in accordance with the principles of the Helsinki Declaration. Approval for the study was granted by the Clinical Research Ethics Committee of Pamukkale University (decision no: 60116787-020/24512, dated: 02.04.2019).

### 2.3. TMS protocol

The TMS treatment protocol was applied using a Neuro-MS/D device (Neurosoft Ltd, Russia) with a figure-of-8 coil. At the first stage, the resting motor threshold was determined based on the involuntary contraction of the fingers on the contralateral hand with gradually increasing stimuli applied 5cm lateral of the vertex of the mid interauricular line. Accordingly, the application severity was defined as 110% of the motor threshold determined. The application site was accepted as 5-cm anterior over the parasagittal plane of the motor cortex point where the motor threshold was determined. This area corresponded to the left dorsolateral prefrontal cortex. The coil was then placed on the scalp at an angle of 45° to the sagittal line. Each TMS session was applied as 25 consecutive trains at 10 Hz frequency, at 40 pulses in each train and the intertrain interval was 20 s. The total of 20 sessions (20,000 pulses) were applied as 1 session per day. The sham-TMS application was performed using the same coil as in the active application but placed at a distance from the scalp at a 45° angle (90° in the sagittal line). Thus, the patients experienced the same sound and sensory effects as in the actual procedure but no stimulation was applied to the cortical structures below the area where the coil was placed [26].

### 2.4. Statistical analysis

The study data were analysed statistically using SPSS v. 25.0 software (IBM SPSS Statistics, IBM Corporation, Armonk, NY, USA). Continuous variables were stated as mean ± standard deviation (SD) values, and categorical variables as number (n) and percentage (%). Conformity of the data to normal distribution was assessed with the Shapiro-Wilk test. In the examinations of independent groups, one-way variance analysis (posthoc: Tukey test) and the Significance of the difference between two means test were applied to variables meeting parametric test assumptions. When variables did not meet the parametric test assumptions, Kruskal-Wallis variance analysis (posthoc: Bonferroni corrected Mann-Whitney U test) and the Mann-Whitney U test were applied. In the comparisons of differences between measurements, the paired samples t-test was applied to parametric data and the Wilcoxon paired samples t-test when parametric assumptions were not met. The chi-square test was applied to examine differences between categorical variables. A value of p < 0.05 was accepted as statistically significant. Bonferroni correction was used to avoid type 1 error, and the level of significance was defined by dividing the p-value (0.05) into the number of pairwise comparisons. Spearman correlation analysis was used to evaluate the relationship between continuous variables.

## 3. Results

### 3.1. Clinical and sociodemographic data

The clinical and sociodemographic data of the study participants are summarised below. Other than a significantly higher level of education and employment rate in the control group than in the patient group, the other sociodemographic data were similar in both groups. No difference was found between the treatment and sham groups in respect of clinical characteristics such as age at first psychiatric presentation, number of depressive episodes, duration of last depressive episode, and history of in-patient treatment (Table 1).

**Figure 1:**
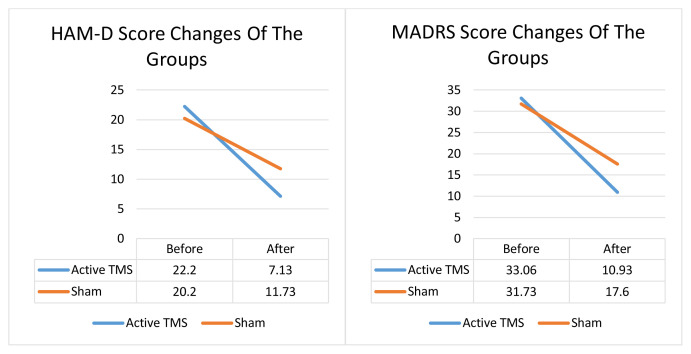
HAM-D and MADRS score changes of the groups. Active TMS Before-After HAM-D p: 0.001; Sham TMS Before-After HAM-D p: 0.001; Active TMS Before-After MADRS p: 0.001; Sham TMS Before-After MADRS p: 0.001.

The mean HAM-D points decreased from 22.2 ± 4.85 to 7.13 ± 3.9 in the treatment group, and from 20.20 ± 4.96 to 11.73 ± 4.75 in the sham group. The mean MADRS points decreased from 33.06 ± 7.28 to 10.93 ± 5.28 in the treatment group, and from 31.73 ± 8.92 to 17.60 ± 5.22 in the sham group. A significant decrease was determined in the MADRS and HAMD scores in both groups (p < 0.05), with a greater difference from pre- to postprocess in the active stimulation group. The mean HAM-D score decreased by mean 15.06 points in the group receiving active stimulation, and by 8.46 points in the sham group. The MADRS mean score decreased by 22.13 points in the active stimulation group, and by 14.13 points in the sham group (Figure 1).

### 3.2. Neurocognitive evaluation data

The results of the cognitive function tests before the procedure are shown in Table 2. The performance of the control group was better than that of the patient groups in the WCST, Stroop test, DST, TMT A-B, and VMPT (p < 0.05). No difference was seen between the active treatment and sham groups in respect of the WCST, Stroop test, DST, TMT A-B, and VMPT (p > 0.05) (Table 2).

**Figure 2:**
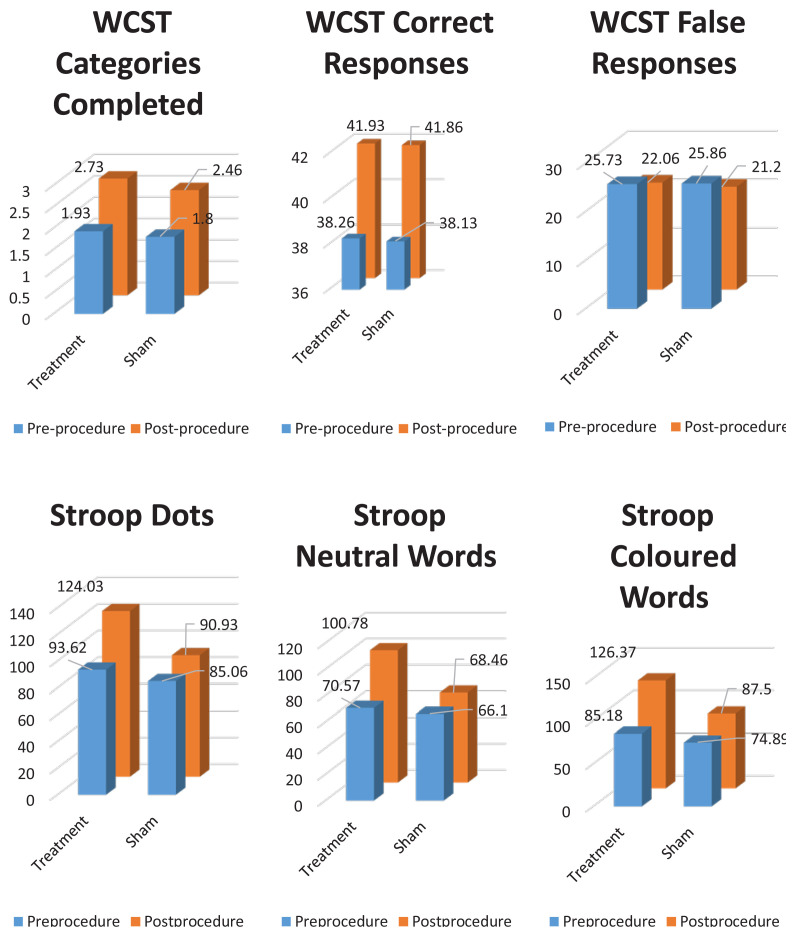
The effects of TMS on cognitive functions. WCST- Categories Completed Treatment p: 0.017, Sham p: 0.083; WCST- Correct Responses Treatment p: 0.046, Sham: 0.085; WCST- False Responses Treatment p: 0.046, Sham: 0.085; Stroop- Dots Treatment p: 0.008, Sham: 0.135; Stroop- Neutral Words Treatment p: 0.003, Sham: 0.565; Stroop- Coloured Words Treatment p: 0.012, Sham: 0.141.

The results of the cognitive function tests after the procedure are shown in Table 3. No significant difference was determined between the patient groups in respect of performance in the WCST, DST, TMT A-B, and VMPT after the procedure (p > 0.05). In the 2nd part of Stroop test, the patients applied with the active stimulation showed a better performance than the patients applied with the sham stimulation (p < 0.05) (Table 3). When the performances within each group were evaluated, the active stimulation group were observed to have significant improvements in the WCST in the number of total number of correct responses, categories completed, and the total number of false responses after treatment compared to pre-treatment (Figure 2). No difference was determined between the pre- and postprocedure WCST performances of the sham group. In the Stroop test, the active treatment group showed significantly better performance in all 3 categories after the procedure (Figure 2).

No significant difference was determined between the pre- and postprocedure results of the DST in both groups. The patient groups showed significantly better performance in the postprocedure tests of the TMT in both section A and section B. In the VMPT, no significant difference was determined between pre- and postprocedure in the treatment group. The instant recall and long-term recall points were determined to be significantly higher postprocedure compared to preprocedure in the sham group (Table 3).

Correlation analyses were performed to distinguish the effects of TMS from the cognitive effects of recovery from depression. No correlation was found between the changes in the depression rating scale and the improving effects on cognition (p> 0.05).

## 4. Discussion

The main outcomes of the current study were that TMS is an effective treatment in treatment-resistant depression, and can provide improvements in cognitive functions. Taking into account the importance of cognitive impairment in predicting the prognosis, life quality, functioning and risk of relapse in depression, the results of this study showed some novel promise.

In this study, in which the efficacy of TMS treatment in treatment-resistant MDD was evaluated, a significant decrease was determined in the MADRS and HAM-D points in both groups after the procedure compared to preprocedure. These results show that TMS also has a significant placebo efficacy. Nevertheless that a greater improvement was seen in the treatment group than in the sham group was similar to the findings of previous metaanalyses that have shown the superiority of TMS treatment to sham TMS [12,14,27,28].

In the majority of studies of TMS treatment that have included patient groups with depression, the mean HAM-D points have been reported to be 19 to 30 [29]. The relatively lower preprocedure scale points in the current study can be attributed to the inclusion of outpatients and the exclusion of more severe patients with any psychotic characteristics and/or at risk of suicide. These conditions could also have contributed to the benefit of the treatment. Previous studies have shown that the majority of patients experiencing a placebo effect from sham TMS are mild-moderate MDD patients [30].

It is known that there are impairments in attention, memory and executive functions in patients with depression [31–34]. Consistent with findings in the literature, cognitive functions of the current study patient group were found to be impaired compared with the control group and positive results were obtained of a partial improving effect on cognitive functions.

The dorsolateral prefrontal cortex (DLPFC) has been shown to be the area responsible for the executive functions of the prefrontal cortex, which are thought to have a significant role in the etiology of MDD [35]. The WCST, which is especially sensitive to DLPFC functions, evaluates the problem-solving ability of an individual and various problem-solving strategies appropriate to different conditions. The current study results showed a significant increase after treatment in the number of categories completed and the total number of correct responses and a significant decrease in the total number of incorrect responses in the group that received the active stimulus to the DLPFC, whereas no difference was determined in the WCST subtests in the group that received sham TMS. These results suggest that TMS could have beneficial effects on executive functions.

The Victoria Stroop test form, which evaluates selective attention, focussed attention, response inhibition, resistance to interference, and information processing speed, was used in this study. According to the mean points of all 3 sections of the test, the patient group showed a worse performance than the control group, but posttreatment, there was observed to be a significant improvement in the group that received the active TMS. These results suggest that in patients with depression, complex attention, mental flexibility, and the skills to be able to resist interference and inhibit response are impaired, and TMS treatment could have positive effects on these abilities. Stroop test is a reliable measurement of top to bottom attention control, including long-term attention and inhibition, and the increase in general performance in the Stroop test that was seen in the current study with the application of rTMS to the DLPFC was consistent with the literature [36–39].

DST is a test which measures the ability to perceive and repeat audio stimuli, and allows interpretation of short-term memory, attention, and working memory. The significantly higher mean DST points of the control group than the patients in the current study showed that instant short-term memory and attention were impaired in the patients with depression. Although there was a decrease in the severity of depression following active TMS treatment compared to the sham group, no significant change was observed in the DST, which suggested that while there was earlier improvement in the numerical areas of cognitive functions, the improvement in audio attention and memory-related functions may occur later.

That an improvement was determined in the sham group compared to the active TMS group in the VMPT was evaluated as a contradictory finding. In addition to showing no significant improving effect on verbal learning and memory processes, these results raise the question of whether TMS could suppress audio attention and memory in the early period. To be able to answer this question, there is a need for further more detailed observational studies of larger samples to investigate the effect of TMS on cognitive functions.

Trail Making test (TMT) is a test which measures executive functions such as complex attention, set changing, and planning, which require visual-mechanical processing skills. The TMT-A evaluates processing speed related to visual scanning ability, and the TMT-B evaluates the ability to switch the setup and follow the sequencing [40]. The patient group values of the TMT A and B sections applied before treatment supported the impairment of executive functions observed in depression. Following TMS the significant increase in performance in both the active and sham stimulus groups in parallel with the improvement in depressive symptoms suggested that the test performance may be associated with the placebo effect.

The effects of TMS on depressive disorder were evaluated in this study with the MADRS and HAM-D scales, and the data showed that TMS was effective on depressive symptoms. However, previous studies that have used the sham TMS protocol at an angle of 45° to minimise the actual stimulus effects have stated that there could be a partial active stimulus which could provide the targeted clinical improvement, indicating that the results could be debatable [41,42]. Although the current study results show a positive change in some cognitive functions, there was seen to be no significant effect in some cognitive areas such as verbal learning and memory.

Cognitive deficits in MDD make a significant contribution to the disabling effects on daily functioning and work performance [2,3]. Authors in this field have reported that the cognitive defects observed in MDD continue even after the elimination of mood and behavioural symptoms [5,6]. In most cases, residual cognitive function symptoms are known to persist like a ‘scar’ after symptom remission even though other depressive symptoms improve [43]. Therefore, a rapid improvement in cognitive functions is not expected in depression treatment. Even so, the effects of TMS were differentiated from the cognitive effects of recovery from depression and some other factors. No correlation was determined between changes in the depression rating scale and improving effects on cognition. Thus, the effects of TMS on cognitive functions are independent from recovery from depression. Moreover, the active stimulation group and sham group were similar in respect of sociodemographic and clinical characteristics that can affect cognitive functions such as age, sex, education, age at first psychiatric presentation, number of depressive episodes, and duration of last depressive episode.

It has also been reported in the literature that cognitive symptoms may be seen before the onset of MDD [3], and there is also evidence even suggesting that cognitive defects seen in healthy individuals could be precursors and predictors of the development of a depressive episode in the future [44,45]. In a prospective, long-term, paired study by Vinberg et al., it was shown that low performance in attention and language functions in addition to executive function defect could predict the onset of the development of depression in the future [46]. Low performance in executive function, attention, and language measurements has been found to be associated with the occurrence of mood disorders and anxiety disorders. It is thought that individuals with better cognitive functioning before the onset of disease are protected against MDD by as yet unknown mechanisms [47]. Delayed correction of cognitive functions may increase the risk of recurrent MDD.

It has been reported that TMS does not have a negative effect on cognitive functions and it is also stated in the literature that TMS has improving effects on cognitive functions [17,48,49]. Moreover, studies have shown that rTMS is possibly effective in improving cognitive function, apathy, language and memory in the early stage of Alzheimer’s disease [50]. In the current study, the active stimulation group showed a better performance than the sham group in cognitive functions with the exception of verbal memory processes. At this point, the importance of this study is that, some interesting findings were determined in the Verbal Memory Processes Test. The improvement in verbal cognitive functions was better in the sham group compared to the active TMS group. The lover rate of improvement seen after active TMS in verbal learning and memory processes therefore raises some questions. Could these results show that TMS does not have a significant improving effect on verbal learning and memory processes? Even more, could TMS have suppressing effects on auditory attention and memory in the early period?

There were some limitations to this study, primarily that because of the effect of the COVID-19 pandemic there were interruptions to the data collection process, some patients left the study before completion and new patients could not be included because of the halting of outpatient TMS protocols. The low number of study participants created a limitation in the comparisons made. Another limitation of the study was the statistically higher education level and employment status of the control group than the patient group. In addition, the effect of TMS alone could not be evaluated as the patients continued pharmacological treatments throughout the study. That a neuronavigation method for localization of the area to which TMS was to be applied and a sham headpiece could not be used because of the high cost entailed also constituted limitations of the study.

In conclusion, the results of the current study demonstrated that with the exception of verbal memory processes, there was a tendency for improvement in the cognitive defects determined in the active phase of resistant depression following TMS treatment, and therefore the clinical use of TMS could provide improvement in cognitive functions in the early stage. However, as lover performance was shown on verbal learning and memory processes in the active stimulation group, these results raise the question of whether TMS could suppress audio attention and memory in the early period.

## Figures and Tables

**Table 1:** Sociodemographic and clinical characteristics of the groups.

	Patients (n: 30) Treatment sham n (%) n (%)		Healthy Control (n: 15) n (%)	p1	p2	p3

**Age (Mean ± SD)**	40.60 ±7.21	37.73 ± 9.33	38.40 ± 8.5	0.682	0.622	0.623

**Sex**						
**Female**	13 (86.6)	9 (60)	10 (66)	0.732	0.245	0.219
**Male**	2 (13.3)	6 (40)	5 (33)			

**Marital status**						
**Married**	8 (53.3)	9 (60)	8 (55.6)	0.493	0.574	0.703
**Single**	6 (40)	3 (20)	5 (31.1)			
**Divorced**	1(6.7)	3 (20)	2 (13.3)			

**Working status**						
**Employed**	9 (60)	6 (40)	15 (100)	0.001	0.745	0.002
**Unemployed**	6 (40)	9 (60)	0 (0)			

**Education status**						
**Primary**	6 (40)	7 (46.7	0 (0)			
**High school**	2 (13.3)	7 (46.7)	7 (46.7)	0.004	0.483	0.002
**University**	7 (46.7)	1 (6.7)	8 (53.3)			

**Disease onset age (Mean ± SD)**	31.93 ± 8.8	29.86 ± 4.8			0.83	

**Depressive episode (n, %)**	2.4 ± 1.2	1.8 ± 0.9			0.75	

**Duration of last episode (month) (Mean ± SD)**	4.9 ± 2.9	5.1 ± 3.2			0.096	

**Hospitalisations**						
**Yes**	9 (60)	4 (26)			0.61	
**No**	6 (40)	11 (74)				

P1: patient-control, P2: treatment group-sham group, P3: treatment group-sham group-control.

**Table 2:** Cognitive functions of the groups before the procedure.

	Patients		Healthy control	p1	p2
	Treatment (Mean ± SD)	Sham (Mean ± SD)			
**Wisconsin Card Sorting Test**					
Number of categories completed	1.93 ± 0.88	1.80 ± 0.86	2.73 ± 1.75	0.106	0.861
Total number of correct responses	38.26 ± 6.79	38.13 ± 7.79	43.06 ± 10.96	0.081	0.999
Total number of false responses	25.73 ± 6.79	25.86 ± 7.79	20.93 ± 10.96	0.081	0.999
Number of perseverative responses	23.00 ± 7.23	21.46 ± 7.67	17.06 ± 8.37	0.151	0.886
Number of perseverative false	13.40 ± 5.93	13.86 ± 8.31	7.93 ± 5.99	0.011*	0.981
Number of nonperseverative false	12.33 ± 7.23	12.00 ± 6.61	13.00 ± 11.14	0.8	0.9
Conceptual number score	32.06 ± 9.49	30.43 ± 9.85	36.66 ± 14.15	0.208	0.945
Learning to learn score	2.67 ± 4.90	0.89 ± 1.45	0.0067 ± 6.51	0.432	0.868
**Stroop Test**					
Chapter 1 (dots)	124.038 ± 72.21	90.933 ± 24.10	75.230 ± 44.6	0.009*	0.603
Chapter 2 (neutral words)	100.788 ± 76.16	68.465 ± 24.38	54.875 ± 23.02	0.016*	0.729
Chapter 3 (coloured words)	126.37 ± 95.24	87.502 ± 44.07	60.254 ± 21.81	0.003*	0.911
**Digit Span Test**					
	6.6 ± 2.06	5.73 ± 1.75	8.06 ± 1.75	0.003*	0.417
**Trail Making test**					
TM A	11.73 ± 4.68	13.78 ± 5.43	22.13 ± 6.30	0.001*	0.571
TM B	9.45 ± 3.76	11.30 ± 3.76	17.78 ± 5.60	0.001*	0.499
**Verbal Memory Process Test**					
Immediate memory	5.06 ± 2.18	3.93 ± 1.66	5.93 ± 1.79	0.024*	0.647
Short-term memory recall	11.93 ± 2.68	11.00 ± 3.29	14.33 ± 1.34	0.001*	0.921
Long-term memory recall	9.26 ± 2.71	8.20 ± 2.59	10.66 ± 2.19	0.021*	0.916
Learning score	65.6 ± 24.78	87.40 ± 28.73	118.13 ± 16.15	0.001*	0.616

P1: patients-healty control, P2: treatment group-sham group,

* Significant at p <0.05.

**Table 3:** Cognitive functions of the groups after the procedure.

The score difference between pre-TMS and post-TMS.	Treatment (Mean ± SD)	Sham (Mean ± SD)	p2
**Wisconsin Card Sorting Test**			
Number of categories completed	0.8	0.66	
**p1**	0.017*	0.083	0.595
Total number of correct responses	3.66	4.73	
**p1**	0.046*	0.085	0.727
Total number of false responses	3.66	4.66	
**p1**	0.046*	0.085	0.743
Number of perseverative responses	2.6	2	
**p1**	0.167	0.316	0.091
Number of perseverative false	1.2	1.06	
**p1**	0.342	0.455	0.943
Number of nonperseverative false	2.46	3.46	
**p1**	0.213	0.126	0.728
Conceptual number score	2.86	5.33	
**p1**	0.243	0.107	0.531
Learning to learn score	0.09	1.15	
**p1**	0.945	0.564	0.613
**Stroop Test**			
Chapter 1 (dots)	30.41	5.87	0.067
**p1**	0.008*	0.135	
Chapter 2 (neutral words)	30.21	2.36	
**p1**	0.003*	0.565	0.045*
Chapter 3 (coloured words)	41.18	12.61	
**p1**	0.012*	0.141	0.067
**Digit Span Test**			
	0.26	0.6	
**p1**	0.157	0.082	0.539
**Trail Making test**			
**TM A**	2.28	2.72	
**p1**	0.001*	0.015*	0.704
**TM B**	1.51	1.98	
**p1**	0.011*	0.028*	0.632
**Verbal Memory Process Test**			
Immediate memory	0.33	1	
**p1**	0.486	0.022*	0.31
Short-term memory recall	0.33	0.13	
**p1**	0.554	0.858	0.613
Long-term memory recall	0.46	1.4	
**p1**	0.277	0.014*	0.159
Learning score	2.73	0.26	
**p1**	0.548	0.952	0.696

P1: preliminary test-posttest, P2: tretament group-sham group,

* Significant at p < 0.05.
